# A Review on the Recent Progress in Matrix Solid Phase Dispersion

**DOI:** 10.3390/molecules23112767

**Published:** 2018-10-25

**Authors:** Xijuan Tu, Wenbin Chen

**Affiliations:** 1 College of Bee Science, Fujian Agriculture and Forestry University, Fuzhou 350002, China; xjtu@fafu.edu.cn; 2 MOE Engineering Research Center of Bee Products Processing and Application, Fujian Agriculture and Forestry University, Fuzhou 350002, China

**Keywords:** sample preparation, matrix solid phase dispersion, sorbent, miniaturization, on-line

## Abstract

Matrix solid phase dispersion (MSPD) has proven to be an efficient sample preparation method for solid, semi-solid, and viscous samples. Applications of MSPD have covered biological, food, and environmental samples, including both organic and inorganic analytes. This review presents an update on the development of MSPD in the period 2015~June 2018. In the first part of this review, we focus on the latest development in MSPD sorbent, including molecularly imprinted polymers, and carbon-based nanomaterials etc. The second part presents the miniaturization of MSPD, discussing the progress in both micro-MSPD and mini-MSPD. The on-line/in-line techniques for improving the automation and sample throughput are also discussed. The final part summarizes the success in the modification of original MSPD procedures.

## 1. Introduction

Sample preparation is the key step in analytical workflow [1]. For solid, semi-solid, and viscous samples, procedures of sample preparation generally start with extracting analytes from matrix into homogeneous liquid solvents. Then a consequent clean-up step may be performed to reduce interference compounds in the extract. Finally, an additional enrichment or concentration step may also be required to meet the sensitivity of the analytical technique. Limitations in the classical method are the use of large volumes of solvent, labor-intensive, and time-consuming through the manipulation. Matrix solid phase dispersion (MSPD), first introduced by Barker et al. [2], provides an alternative approach to reduce solvent use and analysis time for preparing solid, semi-solid, and viscous samples [3].

In a typical MSPD procedure, samples are blended with sorbent to obtain homogeneous mixture. The resulting mixture is transferred and packed into an extraction column. Then solvent is passed through the column to carry out washing and elution step for the extraction and isolation of analytes from the matrix. In some case, an additional co-sorbent could be loaded at the bottom of the column to further clean-up the eluent. Generally, the final extract can be analyzed by chromatography based analytical techniques. Compared with classical solvent extraction method, MSPD eliminates steps of repeated centrifugation and/or filtration, and procedures of re-extraction. Different with solid phase extraction (SPE), in which separated solvent extraction procedure is required to make solid samples suitable for loading into a SPE column, MSPD eliminates the solvent extraction step. These would dramatically reduce the consumption of solvent and the required manipulation time for the preparation. There have been extensive reviews regarding the trends and developments of MSPD [3,4,5,6,7,8,9,10]. In this review, we focus attention on the latest developments in MSPD sorbent, miniaturization of MSPD, on-line/in-line techniques, and the modification of original MSPD procedure. Literatures during 2015 and June 2018 are reviewed to avoid the overlap with recent excellent reviews [9,10].

## 2. Latest Developments in MSPD Sorbent

Molecularly imprinted polymers (MIPs), the synthetic sorbents which exhibit selective binding of target molecular, have been widely used for the extraction of specific compounds [11,12]. In MSPD, sorbent requires to be blended with sample to obtain homogeneous mixture. To improve mechanical strength of MIPs materials, imprinted polymers can be synthesized using other sorbents as carrier. For example, MIPs were prepared on carbon nanotubes (CNTs) for the MSPD preparation of malachite green in aquatic products [13]. Silica gel [14], silica nanoparticles [15], and mesoporous silica [16] also have been reported as the carrier of MIPs to improve the selectivity of MSPD sorbents. Additionally, Wang et al. reported the synthesis of mixed-template MIPs for the extraction of multi-class veterinary drugs [17]. This novel MIPs sorbent was used for the simultaneous MSPD extraction of 20 drugs in meat, including 8 fluoroquinolones, 8 sulfonamides and 4 tetracyclines.

Graphene is one of the carbon-based nanomaterials which shows great promise in sample preparation [18,19]. Graphene provides large surface area and nanosheets morphology for improving adsorption capacity. In addition, the delocalized π electron system in graphene could make it form strong π-stacking interaction with compounds containing aromatic rings. These properties make graphene a good candidate for the adsorption of benzenoid compounds. Sun et al. reported a graphene-encapsulated silica sorbent for the analysis of flavonoids in the leaves of *Murraya panaculata* (L.) Jack [20]. Immobilized on the of surface of silica gel avoided the aggregation and maintained the large surface area and π-electron rich structure graphene during the mechanical blending. Compared with five sorbents (graphene, silica gel, C18, diatomaceous earth, and neutral alumina), graphene-encapsulated silica showed the better extraction efficiency for the target flavonoid compounds.

Phenyltrichlorosilane-functionalized magnesium oxide microspheres were designed by Tan et al. for the extraction of polycyclic aromatic hydrocarbons (PAHs) in soils [21]. This material takes advantage of the high affinity between magnesium oxide and PAHs to enhance the retention of target molecules. Grafting the microspheres with phenyltrichlorosilane reduced the competitive adsorption of chlorine-contained interferences which are widely exist in soil samples. Using hexane and DCM as rinsing and eluting solvent, respectively, seven PAHs were successfully determined in HPLC-FLD with limits of detection (LODs) of 0.02–0.12 µg/kg.

The use of polyethyleneimine (PEI)-modified attapulgite material as MSPD sorbent was reported by Wang et al. for the determination of cadmium in seafood products [22]. Introducing of PEI, which is a cationic polymer with high affinity to cadmium ion, resulted in the high recovery of target ion in complex matrices. High concentration of HNO_3_ (50%, *v*/*v*) was required to release the cadmium. Determined by atomic absorption spectrometry (AAS), the LOD of cadmium in fish sample was found to be 2.5 μg/kg.

Additionally, sorbents such as mussel shell [23,24], molecular sieve [25,26], microcrystalline cellulose [27], and metal-organic framework materials [28,29] also have been reported. These emerging sorbents are summarized in Table 1.

## 3. Miniaturization of MSPD

In classical MSPD protocol, the sample amount is typically 0.5 g [3]. The miniaturization of MSPD (micro/mini-MSPD) can significantly reduce the sample amount, and consequently the consumption of sorbent, solvent, and preparation time. Developed micro/mini-MSPD methods are summarized in Table 2. For instance, Guerra et al. developed a method based on micro-MSPD combined with LC-MS/MS for the simple and rapid determination of dyes in cosmetic products [31]. The proposed micro-MSPD was carried by grounding 0.1 g cosmetic sample with 0.3 g anhydrous Na_2_SO_4_ (drying agent) and 0.4 g of Florisil. After transferring the mixture into a glass Pasteur pipette, 2 mL of methanol was eluted to extract nine water-soluble dyes. By using micro-MSPD method, time and solvent consumption in the sample preparation could be reduced. 

Taking advantage of high sensitive detection methods, sample amount in recently published mini-MSPD could be reduced to the scale of milligram. Chen et al. reported a sensitive quantification of mercury distribution in fish organ based on the mini-MSPD [32]. The sample amount in this research was as low as 1 mg of organ sample. Multiwall carbon nanotubes (MWCNTs) were used as the sorbent, with amount of 0.5 mg. Mercury species were eluted by 100 µL eluent containing HCOOH and l-cysteine. When combined with a sensitive mercury determination method named single-drop solution electrode glow discharge-induced cold vapor generation combined with atomic fluorescence spectrometry, LOD of 0.01 µg/L was achieved. The consumption of sample, adsorbent, and solvent were all dramatically decreased in this mini-MSPD.

Another example of mini-MSPD was reported by Deng et al. [33], in which only 0.30–0.80 mg of plant samples were ground with 2 mg C18 sorbent in liquid nitrogen to obtain the homogenous mixture. Based on this mini-MSPD and the precolumn derivatization coupled with UPLC-MS/MS determination, phytohormone gibberellins were detected with the limits of quantification (LOQs) of 0.54–4.37 pg/mL. As only sub-milligram sample was required for the determination, a spatial distribution of gibberellins in a single *Arabidopsis thaliana* leaf with resolution of 2 × 2 mm^2^ was profiled.

## 4. On-Line/In-Line MSPD

On-line/in-line sample preparation techniques that couple sample preparation step and chromatography separation are regarded as a promising technique with advantages of automatable high sample throughput, reducing sample manipulation and contamination, improving precision, and lower regent consumption [42]. On-line/in-line MSPD provides a potential automated way for the sample preparation of solid, semi-solid, and viscous samples.

Rajabi et al. reported an in-line micro-MSPD method for the determination of Sudan dyes in spices [43]. In this in-line MSPD, the filled MSPD column was placed in the mobile phase pathway before the analytical column. Then the mobile phase passed through the MSPD column to elute analytes and subsequently separated in a reverse-phased HPLC. Since the in-line method integrated extraction and separation into one step, this proposed approach was much faster than other reported methods for the determination of Sudan dyes.

Gutiérrez-Valencia et al. developed an on-line MSPD-SPE sample preparation method combined with HPLC-FLD for the analysis of PAHs in bovine tissues [44]. The bovine liver sample (50 mg) was dispersed on C18 sorbent (200 mg). Then the obtained homogenous mixture was packed into a stainless steel cartridge which was connected to a MSPD-SPE-HPLC-FLD system. The SPE column was used to trap and pre-concentrate the target compounds eluted from the MSPD cartridge. Acetonitrile (ACN)-water mixture and pure ACN solution were applied to wash and elute the MSPD cartridge, respectively. However, ACN extract exhibited poor retention of analytes in C18 SPE column. Thus a dynamic mixing chamber was required to dilute the ACN extract with water before pre-concentration to quantitatively transfer PAHs from MPSD cartridge to the SPE column. Finally, the analytes pre-concentrated on the SPE column were eluted through the guard-column and the analytical column with mobile phase and detected by FLD. Compared with off-line MSPD, the on-line MSPD method showed advantages of lower consumption of sample amount and saving of analysis time.

Additionally, an on-line MSPD-HPLC-ICP-MS method for the determination of mercury speciation in fish was reported by Deng et al. [45]. In this on-line MSPD performance, 1 mg fish sample was blended with 2 mg of MWCNTs, then the mixture was transferred into a stainless steel column which was prior loaded with 0.20 g of C18. The eluent solution containing HCl (2%, *v*/*v*) and l-cysteine (1.5%, *m*/*v*) was loaded by a 100 µL loop through the six-port valve. Then mobile phase flushed the eluent to pass through the MSPD column for the extraction of analytes, which were further separated and detected by HPLC-ICP-MS. It is interesting to notice that the on-line MSPD system consisting of two sequential valves and six stainless steel MSPD columns to improve sample throughput. This on-line system shows the potential of automatable high sample throughput in MSPD method.

## 5. Modification of Original MSPD

**Figure 1:**
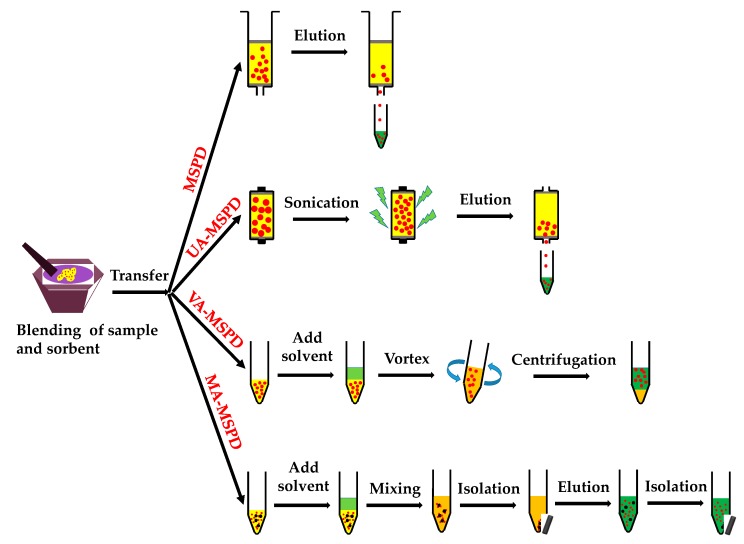
Schematic procedure of original matrix solid phase dispersion (MSPD), ultrasonic-assisted MSPD (UA-MSPD), vortex-assisted MSPD (VA-MSPD), and magnetically-assisted MSPD (MA-MSPD).

The original MSPD can be modified or combined with other extraction methodologies to improve the extraction yields or simplify the MSPD procedures. The schematic procedure of the original and representative modification of MSPD is shown in Figure 1. For instance, ultrasonic-assisted MSPD (UA-MSPD) was first reported by Ramos et al. to improve the extraction yields by putting MSPD column into ultrasonic bath or sonoreactor after the extraction solvent was loaded into the MSPD column [46]. As summarized in Table 3, UA-MSPD has been introduced for the analysis of multi-class organic contaminants. For example, Albero et al. developed an UA-MSPD method for the analysis of 17 emerging contaminants in vegetables [47]. In this modified method, vegetable samples (2 g) was blended with Florisil (4 g) and magnesium sulfate anhydrous (1 g), then the homogenous mixture was transferred into a 20 mL glass column. Extraction solution of 8 mL EtAc:MeOH (9:1, *v*/*v*) containing 3% of NH_4_OH were added to the column. After that, column was sonicated for 15 min in an ultrasonic water bath at room temperature for the extraction. Finally, extract was collected under vacuum manifold. Results indicated that better recoveries were obtained with the assistance of sonication.

Vortex-assisted MSPD (VA-MSPD), in which the step of column elution is replaced by vortex, has been developed to reduce the solvent consumption and analysis time. This simplified MSPD procedure has been found applications in the analysis of phytochemical compounds and organic contaminants (Table 3). For instance, Caldas et al. reported the analysis of antifouling booster biocides in marine sediments by employing VA-MSPD [52]. In the sample preparation procedure, the homogenized mixture of sample and sorbent was added into a centrifuge tube. Then the extraction solvent was added, and the sample was vortexed for 1 min. Finally, the mixture was centrifuged, and the supernatant was collected for the LC-MS/MS analysis. Compared with other extraction methods including ultrasonic extraction, SPE, and microwave extraction, this VA-MSPD exhibited the advantages of shorter extraction time and less solvent consumption.

Another recent progress of the modification is the magnetically-assisted MSPD (MA-MSPD) developed by Fotouhi et al. for the extraction of parabens from breast milks [57]. Modified magnetic nanoparticles were used as the sorbent in the MA-MSPD. Milk sample (200 µL) was blended with poly(indole-thiophene) coated magnetic graphene oxide (MGO@PIT, 50 mg) and drying salt Na_2_SO_4_ (550 mg). After blending, the homogenous mixture was transferred into water solution and mechanically stirred for the adsorption of parabens. Then the MGO@PIT with target compounds were isolated from the solution by magnet. Subsequently, analytes were desorbed from the sorbent with methanol. Compared with the magnetic liquid-solid extraction (MLSE) [58,59], a hot topic of nanomaterials in sample preparation, the major difference between MA-MSPD and MLSE is the manipulation of sample. For MLSE, analytes in solid sample are extracted into the liquid solution prior to the introduction of magnetic sorbent. While in MA-MSPD, the solid sample is blended with magnetic nanoparticles to obtain the homogenous mixture. The similarity of these two methods is the replacement of column packing and elution with simple magnetic isolation. This would simplify the preparation step and reduce the extraction time. More importantly, magnetic nanoparticles have been demonstrated to be reusable in the liquid-solid extraction [58]. Thus MA-MSPD may provide a solution for the reusability of sorbent in MSPD.

Recently, we reported the combination of Soxhlet extraction and MSPD to develop a Soxhlet assisted MSPD (SA-MSPD) method [60,61]. In this modification method, sample was blended with silica gel following the original MSPD protocol and loaded into a column of constant pressure funnel. Then elution solvent was heated and continuous refluxed and passed through the column for the extraction and isolation of flavonoids. By comparing with conventional solvent extraction and Soxhlet extraction method, SA-MSPD showed the higher extraction yield with shorter extraction time and less consumption of solvent. Moreover, the introduction of sorbent into the Soxhlet enabled this classical method to be with clean-up ability. More recently, this SA-MSPD method was further combined with acid-hydrolysis for the quantification of flavonoid aglycones in bee pollen [61]. The acid hydrolysis SA-MSPD procedure accomplished the extraction and hydrolysis of flavonoid glycosides into one step, and provided a more efficient sample preparation method for the quantification of flavonoid aglycones.

## 6. Conclusion Remarks

Application fields of MSPD have been extended from the first reported drug residues in biological tissues to the food and environmental analysis, both for organic and inorganic analytes. Development of new sorbent materials for improving the capacity or selectivity is still the exciting research area in MSPD. One of the drawbacks of MSPD is the reusability of the extraction column. Among the emerging MSPD sorbents, modified magnetic nanoparticles are expected to provide the possibility of reusability. Combining high efficient sorbents with ultra-sensitive analytical technologies, miniaturization of MSPD might be found great interests in the analysis of limited or small size samples. Especially, the mini-MSPD may provide more information on the evolution or the spatial distribution of analytes in the sample matrices. In addition, on-line MSPD has shown the possibility of high-throughput analysis in MSPD. This would also be the trend of automation in MSPD. The modification of the original MSPD appears to be simplified the MSPD procedure and could be help for improving the reproducibility of the manipulation.

## Figures and Tables

**Table 1:** Selected representative studies involving developments in MSPD sorbent.

Sorbent	Analytes	Matrix	MSPD Parameters						Detection	LOD (μg/kg)	LOQ (μg/kg)	Ref.
			Sample Amounts (g)	Sorbent Amounts (g)	Blend Time (min)	Co-Sorbent	Washing Solvent	Elution Solvent				
MIPs	Veterinary drugs	Meat	0.2	0.15	3	0.05 g MIPs	3 mL MeOH/H_2_O (2:8, *v*/*v*)	4 mL MeOH/acetic acid (9:1, *v*/*v*)	UPLC-DAD	0.5–3	1.5–6	[17]
CNTs-MIPs	Malachite green	Aquatic products	0.3	0.2	15	None	4 mL 50% aqueous MeOH	3 mL MeOH-acetic acid (98:2, *v*/*v*)	HPLC-UV	0.7	n.r.	[13]
CNTs-MIPs	Camptothecin	Herb (*Camptotheca acuminate*)	0.1	0.1	5	None	5 mL 10% aqueous MeOH	4 mL MeOH-acetic acid (95:5, *v*/*v*)	HPLC-UV	130 μg/L	n.r.	[30]
Silica gel -MIPs	Degradation products of penicillin	Milk	0.3 mL	0.2	n.r.	None	2 mL DCM	3 mL MeOH-10% acetic acid (9:1, *v*/*v*)	HPLC-UV	40/50	130/170	[14]
SiO_2_-MIP	Acrylamide	Biscuit and bread	0.1	0.15	n.r.	None	1 mL hexane	2.5 mL ACN-MeOH (50:50, *v*/*v*)	HPLC-UV	14.5/16.1	40.5/40.1	[15]
Mesoporous silica-MIPs	Ketoprofen	Powder milk	0.05	0.025	n.r.	None	None	1 mL ACN	HPLC-MS/MS	n.r.	n.r.	[16]
Graphene-encapsulated silica	Flavonoids	Herb (*Murraya panaculata* (L.) Jack)	0.025	0.05	3	None	None	5 mL MeOH	UPLC-UV	4–12 μg/L	10–40 μg/L	[20]
PTS-MgO	PAHs	Soils	0.1	0.1	n.r.	0.05 g PTS-MgO	4 mL hexane	4 mL DCM	HPLC-FLD	0.02–0.12	0.07–0.40	[21]
PEI-attapulgite	Cadmium	Seafood	0.21	0.13	n.r.	None	6 mL H_2_O	8 mL 50%HNO_3_/H_2_O (*v*/*v*)	AAS	2.5	8.3	[22]
Golden mussel shell	Pesticides and PPCPs	Mussel tissue	0.5	0.5	5	None	None	5 mL ethyl acetate	LC-MS/MS	3–30	10–100	[23]
Mussel shell	Booster biocides	Fish tissue	0.5	0.5	5	None	None	5 mL EtOH	LC-MS/MS	1.5/15	5/50	[24]
Molecular sieves	Flavonoids	Fruit peels	0.025	0.025	2.5	None	None	0.5 mL MeOH	UPLC-UV	20–30 μg/L	70–90 μg/L	[25]
Molecular sieve	Sesquiterpenes	Herb (*Curcuma wenyujin*)	0.2	0.2	2.5	None	None	1 mL MeOH	MEEKC	5–34 μg/mL	16–78 μg/mL	[26]
Microcrystalline cellulose	Triterpenoid acids	Herb (loquat leaves)	0.024	0.024	1	None	None	0.2 × 3 mL EtOH	UHPLC-Q-TOF	19.6–51.6	65.3–171.8	[27]
MOFs	Pesticides	Coconut palm	0.25	1	3	None	None	20 mL ACN	HPLC-DAD	10–50	50–100	[28]
MOFs	Pesticides	Peppers (*Capsicum annuum* L.)	0.5	0.35	n.r.	1 g Na_2_SO_4_ + 0.5 g silica	None	10 mL DCM	GC-MS	16.0–67.0	50.3–200.0	[29]

DCM, dichloromethane; CNTs, carbon nanotubes; MIPs, molecularly imprinted polymers; PPCPs, pharmaceutical and personal care products; MOFs, metal-organic frameworks; PTS, phenyltrichlorosilane; PAHs, polycyclic aromatic hydrocarbons; PEI, polyethyleneimine; ACN. acetonitrile. n.r., not reported.

**Table 2:** Selected representative studies using miniaturized MSPD.

Analytes	Matrix	MSPD Parameters						Detection	LOD (μg/kg)	LOQ (μg/kg)	Ref.
		Sample Amounts (g)	Sorbent Amounts	Blend Time (min)	Co-Sorbent	Washing Solvent	Elution Solvent				
Dyes	Cosmetic products	0.1	0.4 g Florisil + 0.3 g Na_2_SO_4_	n.r.	0.1 g Florisil	None	2 mL MeOH	LC-MS/MS	0.01–11	n.r.	[31]
Photoproducts of cosmetic preservatives	Personal care products	0.1	0.4 g Florisil + 0.4 g Na_2_SO_4_	5	0.2 g Florisil	None	1 mL hexane-acetone (1:1, *v*/*v*)	GC-MS/MS	31–170	n.r.	[34]
Flavonoids	Lime fruit	0.05	0.15 g Florisil	1	None	None	0.4 mL [Bmin]BF_4_ aqueous solution (250 mM)	UPLC-UV	4.08/5.04 μg/g	14.01/14.56 μg/g	[35]
Phenolic isomers	Honeysuckle	0.025	0.075 g β-cyclodextrin	2	None	None	0.5 mL MeOH-H_2_O (80:20, *v*/*v*)	UPLC-UV-Q-TOF	1.62–3.33 ng/mL	5.52–11.40 ng/mL	[36]
Inorganic iodine and iodinated amino acids	Seaweed	0.05	0.05 g molecular sieve SBA-15	0.5	None	None	0.4 mL [C12mim] Br (200 mM)	UHPLC-UV	3.7–16.7 ng/mL	12.4–55.8 ng/mL	[37]
Phenols	Olive fruits	0.05	0.025 g chitosan	1	None	None	0.5 mL × 3 MeOH-H_2_O (6:4, *v*/*v*)	UHPLC-Q-TOF	69.6–358.4	232–1240.8	[38]
Mercury species	Fish organs	1 mg	0.5 mg MWCNTs	5	0.15 g C18	None	0.1 mL × 2 0.5% l-cysteine and 4% HCOOH	AFS	0.01	n.r.	[22]
Gibberellins	Plant	0.3–0.8 mg	2 mg C18	n.r.	None	None	0.2 mL ACN	UPLC-MS/MS	0.16–1.31 pg/mL	0.53–4.37 pg/mL	[33]
Synthetic dyes	Cosmetics and foodstuffs	0.1	0.4 g C18 + 0.3 g Na_2_SO_4_	n.r.	0.1 g C18	None	2 mL MeOH	LC-MS/MS	14.2–95.2	n.r.	[39]
Phenolic acids	Plant preparation (Danshen tablets)	0.024	0.024 g graphene nanoplatelets	1	None	None	0.2 mL H_2_O	UHPLC-ECD	1.19–4.62 ng/mL	3.91–15.23 ng/mL	[40]
Lignans	Herbs (Schisandrae Chinensis Fructus)	0.025	0.05 g molecular sieve TS-1	2.5	None	None	0.5 mL MeOH	MEEKC	n.r.	2.77 μg/mL	[41]

MWCNTs, multiwall carbon nanotubes; AFS, atomic fluorescence spectrometry. n.r., not reported.

**Table 3:** Selected representative studies using ultrasonic assisted MSPD and vortex assisted MSPD.

Modification	Analytes	Matrix	MSPD Parameters					Detection	LOD (μg/kg)	LOQ (μg/kg)	Ref.
			Sample Amount (g)	Sorbent Amount	Grind Time (min)	Extraction Time (min)	Elution Solvent				
Ultrasonic assisted	Emerging organic contaminants	Poultry manure	0.5	2 g Florisil + 1 g MgSO_4_	n.r.	15	8 mL ACN with 3% NH_4_OH + 10 mL ACN with 4% formic acid	GC-MS/MS	0.9–2.2	2.8–5.5	[48]
Ultrasonic assisted	Emerging contaminants	Vegetables	2	4 g Florisil + 1 g MgSO_4_	n.r.	15	8 mL EtAc:MeOH (9:1, *v*/*v*) containing 3% NH_4_OH	GC-MS/MS	0.1–0.4	n.r.	[47]
Ultrasonic assisted	Emerging contaminants	Aquatic plants	1	4 g Florisil + 2 g MgSO_4_	5	15	8 mL EtAc with 3% NH_4_OH	GC-MS	0.3–2.2	1.0–6.7	[49]
Ultrasonic assisted	Aflatoxins	Rice	1	1 g C18	5	11	4 mL ACN	HPLC-FLD	0.04–0.14 ng/g	0.12–0.56 ng/g	[50]
Vortex assisted	5-HMF and iridoid glycosides	Herb (*Fructus Corni)*	0.02	0.04 g silica	3	3	6 mL [Domin]HSO_4_	UHPLC-UV	0.02–0.08 μg/mL	0.07–0.24 μg/mL	[51]
Vortex assisted	Booster biocides	Marine sediments	2	0.25 g C18	n.r.	1	10 mL MeOH	LC-MS/MS	n.r.	0.5–5	[52]
Vortex assisted	Phenol	Herb (*Forsythiae Fructus*)	0.02	0.02 g Florisil	3	2	2 mL10% (*v*/*v*) Triton X-114	UHPLC-UV	0.03–0.08 μg/mL	0.08–0.25 μg/mL	[53]
Vortex assisted	Ibuprofen enantiomers	Milk	0.5	0.30 g diatomaceous earth + 0.30 g Na_2_SO_4_ + 0.26 g PSA + 0.021 g β-cyclodextrin	5	1	2 mL MeOH	HPLC-UV	0.042/0.045 μg/g	0.14/0.15 μg/g	[54]
Vortex assisted	Pesticides	Drinking water treatment sludge	1.5	0.5 g Chitin	5	1	5 mL ethyl acetate	GC-MS	n.r.	5–500	[55]
Vortex assisted	Pharmaceuticals	Fish tissue	0.5	0.5 g diatomaceous earth + 0.5 g Na_2_SO_4_	5	1	5 mL MeOH	LC-MS/MS	1.5–300	5–1000	[56]

5-HMF, 5-hydroxymethy furfurol. n.r., nor reported.
